# Persistent Post-traumatic Upper Limb Pain and Paraesthesia Following a Fall on an Outstretched Hand: A Case of Inconclusive Localisation

**DOI:** 10.7759/cureus.115575

**Published:** 2026-09-01

**Authors:** Emily R Kemp, Aidan G Ginns

**Affiliations:** 1 Psychiatry, The Rotherham NHS Foundation Trust, Rotherham, GBR; 2 Physiotherapy, Woodland Hospital, Kettering, GBR

**Keywords:** brachial plexus injury, case report, fall on an outstretched hand, radiculopathy, thoracic

## Abstract

Persistent upper limb pain following trauma can present a diagnostic challenge, particularly when clinical findings and investigations are discordant. We report the case of a 31-year-old female patient with persistent right upper limb and anterior chest wall pain associated with sensory disturbance in a multi-regional distribution, following a fall on an outstretched hand (FOOSH). Five years after the initial injury, the patient's symptoms, pain severity, functional limitations and neurological findings remained unchanged despite extensive conservative and interventional management, including physiotherapy, pharmacological treatment, corticosteroid injections, manipulation under anaesthesia and specialist multidisciplinary review. Magnetic resonance imaging (MRI) demonstrated no structural abnormality or nerve root compression sufficient to explain the patient’s presentation, while electromyography (EMG) demonstrated findings suggestive of a mild T1 radiculopathy. These findings were not fully concordant with the clinical presentation. Clinicians should consider a broad differential diagnosis and carefully correlate clinical findings with imaging and electrodiagnostic studies when evaluating persistent post-traumatic upper limb pain.

## Introduction

Upper limb pain following trauma encompasses a broad range of pathologies, and accurate anatomical localisation can be diagnostically challenging because of overlapping sensory distributions involving the cervical and thoracic nerve roots and the brachial plexus [1,2]. This is particularly true when clinical and investigative findings are discordant [3-6]. Thoracic radiculopathy is an uncommon and frequently under-recognised cause of spinal pain syndromes [7,8], with T1 radiculopathy being particularly rare [9]. Although thoracic radiculopathies typically present with band-like thoracic or truncal pain [8], T1 radiculopathy may present with upper-limb symptoms, including pain, weakness, sensory disturbance and reduced reflexes in a distribution corresponding to the affected nerve root [3,4,7], because the T1 nerve root contributes to the lower trunk of the brachial plexus [1,2,8]. Reported causes include thoracic disc disease and systemic conditions such as diabetes mellitus [7-9]. Fall on an outstretched hand (FOOSH) is a common mechanism of upper limb injury and is most frequently associated with distal pathology [10]. However, higher-energy mechanisms may also result in more proximal neural injury [11], with trauma being one of the most common causes of brachial plexopathy [1,11]. Correlation between history, examination, imaging and electrodiagnostic studies is often required, although these findings may not always be concordant [3-6]. We report a case of persistent right upper limb and thoracic wall symptoms following a FOOSH injury, in which discordance between clinical assessment and investigative findings resulted in ongoing diagnostic uncertainty and highlighted the challenges of anatomical localisation after trauma.

## Case presentation

This case describes a 31-year-old right-hand-dominant woman who developed right-sided pain and sensory disturbance following a FOOSH with subsequent impact to the lateral aspect of the right shoulder and upper arm.

The patient described a low-energy mechanism of injury following loss of balance during an evening out. While wearing high-heeled footwear and under the influence of alcohol, she tripped on an uneven surface and fell forwards, sustaining the reported trauma. She immediately experienced severe pain involving the right shoulder, proximal upper arm, anterior chest wall, scapular region and axilla (Figure 1). She also reported paraesthesia involving the anterior shoulder, proximal upper arm, axilla, anterior chest wall, medial arm and all digits except the thumb. Functional allodynia was reported during activities of daily living, including dressing, hair brushing and lying supine. Pain was rated using the Numeric Pain Rating Scale (NPRS), an 11-point self-reported scale ranging from zero (no pain) to 10 (worst imaginable pain) [12]. She reported pain scores of seven on waking, five during the day and eight at night. Symptoms frequently disturbed sleep and were aggravated by lying on the right side. Ice application provided partial relief.

**Figure 1:**
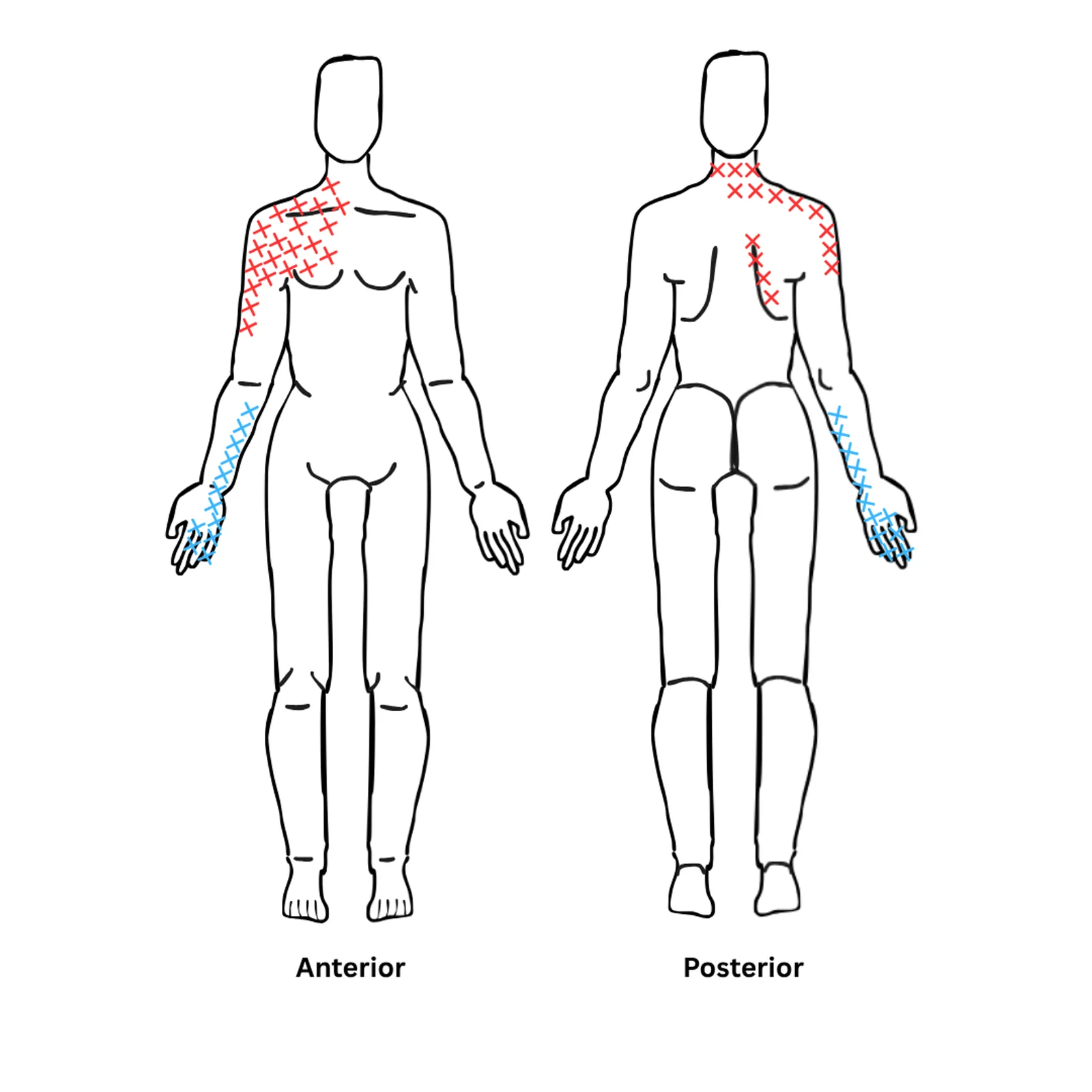
Distribution of pain and sensory disturbance following the initial injury Red indicates areas of reported pain, while blue indicates areas of hyperaesthesia.

The patient did not initially seek medical attention following the injury and independently self-managed her symptoms with simple analgesia. As her symptoms failed to improve, she first presented to her general practitioner (GP) 12 months after the initial injury, who subsequently referred her to orthopaedic services and for physiotherapy. Despite ongoing multidisciplinary input, she continued to experience persistent symptoms and underwent multiple episodes of assessment and treatment across both specialities over the subsequent five years.

The patient was initially reviewed by physiotherapy 16 months after the injury. Observation demonstrated right shoulder depression and anterior protraction at rest, with reduced cervical lordosis and marked thoracic hyperkyphosis. No ptosis, facial asymmetry or cranial nerve abnormalities were identified. Hyperhidrosis was present but reported as longstanding and unchanged from the patient’s baseline. There was no wasting or atrophy of the thenar or hypothenar eminences bilaterally, and no Gilliatt-Sumner hand was observed. Scapular winging was not present. Supraclavicular tenderness was noted, together with tenderness over the C4-T3 spinous processes and associated paraspinal musculature. Hyperaesthesia on light touch was reported on the anterior shoulder, proximal upper arm, axilla, anterior chest wall, medial arm and all digits except the thumb (Figure 1). These findings did not conform to a single peripheral nerve distribution or classical dermatomal pattern. Biceps, brachioradialis and triceps reflexes were symmetrical and preserved bilaterally. Peripheral vascular assessment demonstrated capillary refill of less than two seconds, with normal colour and temperature of the right upper limb. Adson's and Roos' tests were negative. Muscle strength was assessed using the Medical Research Council (MRC) muscle strength grading scale (Oxford scale), with grades ranging from zero (no visible contraction) to five (normal strength) [13]. Testing demonstrated pain-limited weakness rather than clear focal neurological weakness. Shoulder elevation, shoulder abduction and finger flexion were graded four. The left upper limb was normal at five, and strength was five for right-sided elbow flexion and extension, wrist flexion and extension and finger abduction and adduction. Further provocative neurological and musculoskeletal testing was limited by pain; therefore, repeated or more forceful pain-limited testing was not performed to avoid exacerbating the patient's symptoms. Upper limb tension testing, clavicular mobilisation and first rib assessment were also limited by pain and could not be reliably completed. Increased muscle tone was noted within the right deltoid, pectoralis major and biceps brachii muscles. Grip strength was assessed using a North Coast Medical handheld dynamometer, with the shoulder in a neutral position, the elbow flexed to 90 degrees and the forearm maintained in a neutral position, in accordance with the standardised testing position. A one-repetition maximum test was performed for each hand, with findings presented in Table 1. The reduced grip strength was considered likely to reflect pain inhibition rather than true neurological weakness, consistent with the clinical examination findings. Range of active motion measurements for the cervical spine and shoulder are presented in Table 2 and Table 3, respectively, and special test results are reported in Table 4. Movements were measured using a goniometer. Red-flag symptoms were excluded during the assessment.

**Table 1:** Grip strength Reference values for hand grip strength were adapted from Mathiowetz et al., who established age- and sex-specific normative values using dynamometer measurements in healthy adults [14].

Side	Patient	Reference values (mean ± SD)	Interpretation
Right	20 kg	35.7 kg ± 8.7 kg	Reduced
Left	26 kg	30.8 kg ± 8.0 kg	Normal

**Table 2:** Cervical spine range of motion L = left, R = right Reference values for cervical range of motion were adapted from Niewiadomski et al. [15].

Movement	Patient	Reference values (mean ± SD)	Interpretation
Flexion	70°	65.3° ± 12.2°	Normal
Extension	30°	60.9° ± 9.2°	Reduced
Lateral flexion (R)	30°	43.3° ± 12.2°	Reduced
Lateral flexion (L)	40°	45.3° ± 9.2°	Normal
Rotation (R)	50°	71.2° ± 8.1°	Reduced
Rotation (L)	75°	74.5° ± 8.1°	Normal

**Table 3:** Shoulder range of motion L = left, R = right Reference values for active shoulder range of motion were adapted from Gill et al., who reported age- and gender-stratified normative values in a community-based cohort [16].

Movement	Right	Left	Right shoulder reference values (mean ± SD)	Left shoulder reference values (mean ± SD)	Interpretation
Flexion	90°	170°	164.4° ± 13.1°	162.9° ± 12.2°	R = reduced; L = normal
Abduction	70-80°	170°	156.0° ± 12.7°	155.9° ± 12.9°	R = reduced; L = normal
External rotation (neutral)	50°	50°	65.1° ± 13.8°	61.9° ± 14.3°	R = reduced; L = normal

**Table 4:** Special clinical tests

Test	Result	Interpretation
Tinel’s test	Sign was not elicited at the carpal tunnel, cubital tunnel, proximal forearm and dorsal wrist	No peripheral nerve irritation detected
Phalen’s test	Negative	No median nerve compression at carpal tunnel
Durkan’s compression test	Negative	No evidence of carpal tunnel syndrome
Spurling’s test	Reproduced ipsilateral neck and shoulder pain, no radicular symptoms reproduced	Suggestive of cervical root irritation
Hoffman’s sign	Negative bilaterally	No upper motor neuron signs
Tromner’s sign	Negative bilaterally	No upper motor neuron signs

Investigations were undertaken throughout the clinical course. Seventeen months after the initial injury, magnetic resonance imaging (MRI) of the right shoulder demonstrated mild subacromial-subdeltoid bursitis with preservation of the rotator cuff. A cervical MRI performed during the same month identified a small central C6-C7 disc bulge without foraminal stenosis or nerve root compression (Figure 2). The brachial plexus was not assessed. Routine blood tests were unremarkable (Table 5). Repeat shoulder MRI, 41 months after initial injury, demonstrated moderate degenerative change within the sternoclavicular joint without other significant pathology. At a similar time, further cervical and thoracic MRI examinations showed minimal spondylotic change without significant neural impingement, spinal cord abnormality or structural lesion to explain the patient's symptoms. Electromyography (EMG) and nerve conduction studies (NCS) identified findings consistent with a mild subacute right T1 radiculopathy, with the complete results demonstrated in Table 6 and Table 7, respectively. A subsequent ultrasound (US) scan 54 months after initial injury demonstrated rotator cuff tendinopathy and associated subacromial bursitis, as shown in Figure 3.

**Figure 2:**
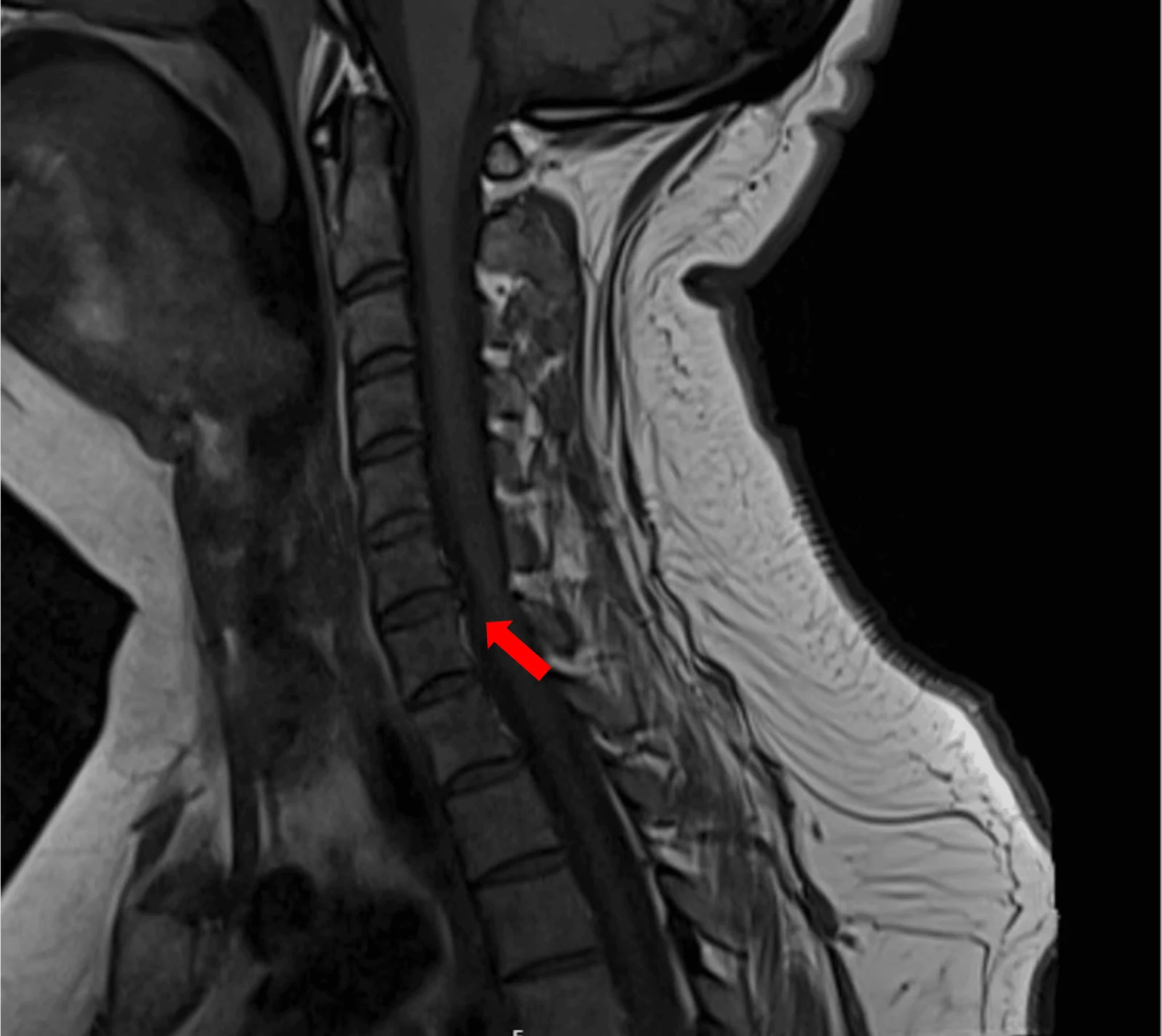
T1-weighted, sagittal MRI of the cervical spine showing C6-C7 central disc bulge but no nerve root compressive lesion This image is included to document the absence of compressive cervical pathology. Although the disc bulge is non‑compressive and not clinically explanatory, its presence confirms that no structural cervical lesion is contributing to the patient’s sensory disturbance.

**Table 5:** Routine blood tests performed one year and four months after initial injury Reference ranges represent the laboratory-specific normal ranges provided by Ramsay Health Care and were obtained from the electronic laboratory reporting system.

Test	Result	Normal values	Interpretation
Haemoglobin	143 g/L	115-155 g/L	Normal
Platelets	264 ×10^9^/L	150-400 ×10^9^/L	Normal
White cell count	7.48 ×10^9^/L	3.0-10.0 ×10^9^/L	Normal
Neutrophils	4.58 ×10^9^/L	2.0-7.5 ×10^9^/L	Normal
Sodium	138 mmol/L	133-146 mmol/L	Normal
Potassium	4.3 mmol/L	3.5-5.3 mmol/L	Normal
Urea	3.2 mmol/L	2.5-7.8 mmol/L	Normal
Creatinine	52 μmol/L	45-84 μmol/ L	Normal

**Table 6:** Electromyography study summary of the right upper limb

Muscle	Insertional activity	Fibrillation potentials	Positive sharp waves	Polyphasic motor units	Duration	Amplitude	Recruitment
Infraspinatus	Nil	Nil	Nil	Normal	Normal	Normal	Normal
Biceps (long head)	Nil	Nil	Nil	Normal	Normal	Normal	Normal
Brachioradialis	Nil	Nil	Nil	Normal	Normal	Normal	Normal
Triceps	Nil	Nil	Nil	Normal	Normal	Normal	Normal
Extensor carpi ulnaris	Nil	Nil	Nil	Normal	Normal	Normal	Normal
First dorsal interosseous	Nil	Nil	Nil	Moderately increased	Normal and long	Normal	Sub-maximal
Abductor pollicis brevis	Nil	Nil	Nil	Normal	Normal	Normal	Normal

**Table 7:** Sensory and motor nerve conduction study summary

Stimulation site	No response	Onset (ms)	Onset-peak amplitude (µV)	Peak-trough amplitude (µV)	Distance (cm)	Velocity (m/s)
Left antebrachial cutaneous sensory (elbow)						
Medial	-	1.8	2.7	5.3	13.0	72
Medial (repeat)	-	1.7	0.6	3.8	13.0	76
Lateral	No response	-	-	-	12.0	-
Right antebrachial cutaneous sensory (elbow)						
Medial	-	2.1	6.3	6.5	13.5	64
Lateral	No response	-	-	-	12.5	-
Lateral (repeat)	No response	-	-	-	12.5	-
Right sensory (wrist)						
Median D2	-	2.4	18.4	20.9	12.5	52
Ulnar D5	-	1.7	9.0	8.4	11.5	68
Radial	-	1.5	12.7	10.4	8.5	57
Right median motor						
Wrist	-	3.9	3.9	7.0	-	-
Right ulnar motor						
Wrist	-	2.9	7.3	11.9	-	-
Below elbow	-	6.8	7.0	10.8	21.5	55
Above elbow	-	8.7	6.4	10.0	11.0	58

**Figure 3:**
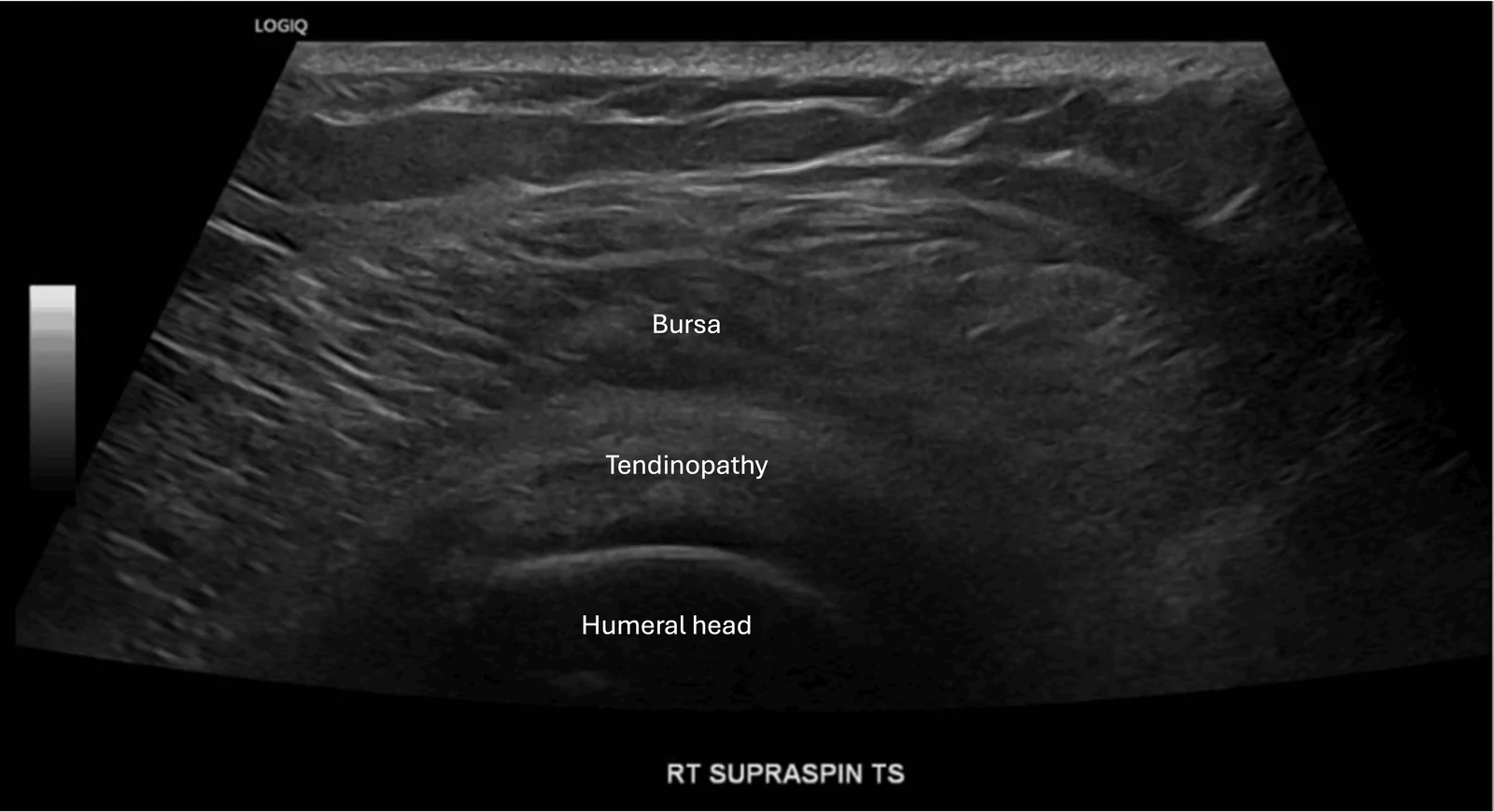
Ultrasound scan of the right shoulder demonstrating rotator cuff tendinopathy and associated subacromial bursitis

The patient underwent multiple conservative and interventional treatment modalities throughout the clinical course, including manipulation under anaesthesia with subacromial corticosteroid injection, physiotherapy, sternoclavicular joint injections, referral to spinal surgery, pain‑management pharmacotherapy and trigger‑point injections to the shoulder, neck and anterior chest wall. Despite these interventions, the patient experienced no sustained improvement in pain, function, sleep, range of motion and sensory symptoms five years after initial injury. A detailed chronological summary of all treatments is provided in Table 8.

**Table 8:** Timeline of clinical assessment, investigations and interventions following the initial injury

Time post-injury	Outcome
Day 0	Tripped and fell, sustaining a low-energy impact to the right shoulder. Immediate onset of right-sided pain and sensory disturbance.
16 months	Physiotherapy assessment due to persistent symptoms. Referred to orthopaedic services.
17 months	First orthopaedic consultant assessment. Right shoulder and cervical spine MRI requested.
20 months	Orthopaedic follow-up and MRI review. Referred for intervention.
24 months	Right shoulder manipulation under anaesthesia with subacromial space and sternoclavicular joint injections. Continued physiotherapy.
25 months	Persistent symptoms despite physiotherapy; referred back to orthopaedics.
27 months	Ultrasound-guided subacromial space injection. Persistent symptoms despite physiotherapy; re-referred to orthopaedics.
33 months	Bilateral sternoclavicular joint injections.
41 months	Discharged from physiotherapy following some improvement in movement and pain, which was not sustained. Minimal overall change from initial presentation. Spinal team review; further MRI and electrodiagnostic studies requested.
45 months	Pain management review, including medication optimisation, workplace adjustments, pacing and postural advice.
52 months	Right shoulder and chest wall trigger-point injections with minimal benefit. Referred to upper limb and hand surgery.
54 months	Ultrasound of the right shoulder requested by orthopaedics.
56 months	Discharged from pain management on nefopam, baclofen and amitriptyline.

## Discussion

Persistent upper limb pain and paraesthesia following trauma present a broad differential diagnosis. In this case, the differential diagnoses considered included cervical and thoracic radiculopathy, brachial plexopathy, thoracic outlet syndrome (TOS), occult fracture, peripheral nerve injury and complex regional pain syndrome (CRPS). Broader contributing concepts, including central sensitisation and psychosocial factors, were also considered.

Radiculopathy has a range of potential aetiologies, including degenerative or herniated discs, space-occupying lesions including malignancy, infection, traumatic or pathological fractures and inflammatory disorders, all of which may result in irritation or compression of the spinal nerve roots [7,8,17]. Thoracic radiculopathy itself is relatively uncommon in clinical practice [7,8], largely due to the structural stability of the thoracic spine provided by the rib cage [7]. Cervical and thoracic radiculopathy were both considered given the upper limb and chest wall symptoms; however, the sensory disturbance did not conform to a single cervical or thoracic dermatome. Axillary and medial upper limb involvement is of particular relevance given the sensory distribution of the intercostobrachial nerve (T2), which communicates with the lower brachial plexus [2,18]. Anatomical variation, including direct T2 contributions to the brachial plexus or additional T1 or T3 contributions to the intercostobrachial nerve, may also be relevant [2,18,19].

MRI and EMG remain the principal investigations used in suspected nerve root pathology. MRI provides information on structural damage, whereas EMG offers a functional evaluation [4,20]. In this case, cervical MRI demonstrated no significant nerve root compression, while EMG findings were suggestive of mild T1 radiculopathy, which reduced support for isolated cervical radiculopathy as the primary diagnosis. The diagnostic accuracy of MRI in radiculopathy remains controversial, with reported limitations in sensitivity and correlation with clinical symptoms [4,5,17,21]. This discordance could reflect the inherent limitations of static imaging, which does not account for dynamic, posture-dependent or non-compressive mechanisms of nerve root dysfunction [4,21]. EMG also has recognised limitations, including variable sensitivity despite high specificity and reduced ability to detect early or purely sensory radiculopathy [3,4,6]. Radecki et al. described a comparable case with similar presentation and conflicting investigative findings [9], highlighting that these modalities assess different aspects of pathology and underscoring the importance of their complementary use alongside clinical assessment in establishing an accurate diagnosis [3,4,20].

A lower trunk brachial plexus lesion was also considered due to the clinical presentation but not confirmed. The T1 nerve root contributes significantly to the lower trunk of the brachial plexus [2], and lesions at this level may produce sensory disturbance that overlaps with T1 radiculopathy on electrodiagnostic studies, limiting precise anatomical localisation [3,9,20]. Furthermore, Choi et al. report that thoracic radiculopathy is often reported to present without clinically significant motor deficit, which may account for the absence of weakness despite electrophysiological evidence of radiculopathy [8]. TOS may also produce a similar distribution of symptoms due to compression of the lower brachial plexus within the scalene triangle and costoclavicular space, where it lies in close proximity to the first rib [22]. However, the minimal positional exacerbation of symptoms and lack of vascular features made TOS a less likely diagnosis [22]. This anatomical continuity between the nerve roots, brachial plexus and thoracic outlet region highlights the potential for overlapping clinical syndromes and supports consideration of lower trunk brachial plexus involvement [2,19,22].

The mechanism of injury is important in contextualising these findings. Bartusek et al. described a range of FOOSH-related pathology, most commonly involving distal upper limb structures such as bone, ligaments, cartilage and other soft tissue [10]. Proximal upper limb injury is less commonly associated with FOOSH; however, the additional direct impact onto the lateral shoulder introduces a plausible mechanism for traction injury across the brachial plexus and cervicothoracic nerve roots [20]. The lower trunk may be particularly vulnerable to traction due to its anatomical relationship with the first rib, a region susceptible to mechanical stress and compression [19,22]. Occult fractures of the clavicle or first rib may also contribute to neuropathic symptoms through neural irritation; however, no radiological evidence of fracture was identified in this case [10,11].

Finally, the delayed presentation and chronicity of symptoms may have contributed to diagnostic complexity. Persistent pain and sensory disturbance over a prolonged period can lead to central sensitisation, a mechanism characterised by amplification of pain processing within the central nervous system, often manifesting as hyperalgesia, allodynia and enhanced central processing of sensory stimuli [23,24]. Similarly, CRPS is an important consideration in the broader differential diagnosis of chronic post-traumatic pain, particularly where disproportionate pain is accompanied by sensory, motor and autonomic disturbances [25]. These mechanisms and conditions represent general considerations in the assessment of persistent post-traumatic pain and were not established as diagnoses in this case. Psychosocial factors, including pain catastrophising and fear-avoidance behaviours, have also been associated with reduced function and increased pain-related disability [24]. Radecki et al. described a case where activities of daily living were modified to avoid painful movements [9]. In the present case, reduced shoulder range of motion, decreased grip strength and compensatory trunk movement during functional tasks may similarly reflect adaptations to persistent pain, although the underlying mechanisms cannot be determined from the available clinical findings.

## Conclusions

This case demonstrates an atypical post-traumatic upper limb sensory syndrome. Although the presentation raised concern for cervical and thoracic nerve root or lower brachial plexus involvement, the findings remained anatomically unlocalised. The co-existence of subacromial-subdeltoid bursitis and cervical degenerative changes further highlights the multifactorial nature of the presentation. This case illustrates the challenges of interpreting discordant investigations in the context of complex post-traumatic upper limb pain and highlights the importance of maintaining a broad differential diagnosis while continuing to investigate the underlying cause. In the absence of a definitive diagnosis, management remains focused on addressing the patient's symptoms and functional limitations with input from specialist teams, including pain management and upper limb and hand surgeons. 
